# Two Cerebrospinal Fluid (CSF) Diversion Procedures for Two Separate CSF Pathologies in a 19-Year-Old Male: A Case Report

**DOI:** 10.7759/cureus.83666

**Published:** 2025-05-07

**Authors:** Saif A Badran, Aous Mohammad Qasim, Bashar Ayad Saeed, Mohammed Thakir Ismail, Mohammed Ali Taher

**Affiliations:** 1 Department of Surgery, Ibn Sina University of Medical and Pharmaceutical Sciences, Baghdad, IRQ; 2 Department of Surgery, College of Medicine, Ninevah University, Mosul, IRQ

**Keywords:** aqueductal stenosis, csf pathology, endoscopic third ventriculostomy, hydrocephalus management, intracranial hypertension

## Abstract

Hydrocephalus is a dynamic neurosurgical entity, with cerebrospinal fluid (CSF) diversion methods continually evolving to strike a balance between long-term efficacy and the risk of complications. We describe a 19-year-old man who was treated with endoscopic third ventriculostomy (ETV) for congenital aqueductal stenosis, with initial relief of symptoms. Two years later, he experienced recurring headaches and papilledema, but neuroimaging showed stable ventricles and patency of the ETV stoma. Magnetic resonance venography (MRV) ruled out venous sinus thrombosis, while lumbar CSF manometry revealed persistently elevated intracranial pressure (ICP), consistent with CSF absorption dysfunction. Given his refractory symptoms despite medical therapy, a lumboperitoneal (LP) shunt was placed, leading to complete resolution of symptoms and papilledema.

This case underscores the paradox of post-ETV intracranial hypertension, reinforcing that a patent ETV does not equate to normal CSF dynamics. Integrating continuous ICP monitoring, advanced CSF flow imaging, and personalized diversion strategies may enhance long-term outcomes in this complex subset of hydrocephalus patients.

## Introduction

Cerebrospinal fluid (CSF) physiology is regulated by production, circulation, absorption, and pulsatile flow, under the control of cerebral compliance, vascular pulsations, and glymphatic clearance [1]. Although conventionally characterized as being unidirectional, new evidence identifies paravascular routes and pressure-driven absorption, contradicting the traditional model [1].

Hydrocephalus, once dichotomized into communicating and non-communicating forms, is now recognized as a spectrum of impaired compliance and CSF outflow resistance [2]. The Marmarou model emphasizes the role of pressure-volume compensation, particularly in cases where post-endoscopic third ventriculostomy (ETV) intracranial hypertension arises from CSF malabsorption rather than obstruction, necessitating alternative diversion strategies [2]. Advances in phase-contrast MRI, infusion studies, and computational fluid dynamics enable precise differentiation of true hydrocephalus from atrophy and idiopathic intracranial hypertension [1,2]. Optimizing CSF diversion strategies requires an individualized approach, integrating pulsatility, compliance, and venous outflow dynamics to improve long-term outcomes [1,2]. Congenital aqueductal stenosis leads to non-communicating hydrocephalus, often managed with ETV as a first-line intervention [3]. However, persistent intracranial hypertension despite a patent ETV stoma suggests secondary CSF absorption dysfunction, a phenomenon poorly understood but linked to impaired glymphatic clearance, altered venous outflow, and arachnoid granulation dysfunction [3]. Computational models identify barrier dysfunction instead of drainage failure as a major cause of retained CSF, perhaps as a mechanism for post-ETV intracranial hypertension necessitating further diversion procedures [4].

Management of hydrocephalus is not easy, with long-term diversion problems comprising ETV failure, impaired CSF absorption, and dependence on the shunt [5]. Persistent intracranial hypertension following ETV with a patent stoma indicates changed compliance mechanics [5]. Shunt dependency is fraught with high rates of failure, with 50% needing revision within five years [6]. Adjustable valve advances in shunts, telemetric monitoring of intracranial pressure, and personalized approaches to diversion are vital in preventing complications and ensuring optimal long-term outcomes [5,6].

This case highlights a rare scenario where a patient required two distinct CSF diversion procedures for separate pathologies, i.e., ETV for obstructive hydrocephalus due to aqueductal stenosis, followed by LP shunting for persistent intracranial hypertension despite a patent ETV. The paradoxical elevation of intracranial pressure post-ETV, in the absence of hydrocephalus, suggests secondary CSF absorption dysfunction. Complying with the CARE (Case Report) guidelines [7], this report emphasizes the dynamic nature of CSF, highlighting the necessity of long-term monitoring and tailored neurosurgical approaches in treating changing intracranial pressure disorders.

## Case presentation

**Figure 1:**
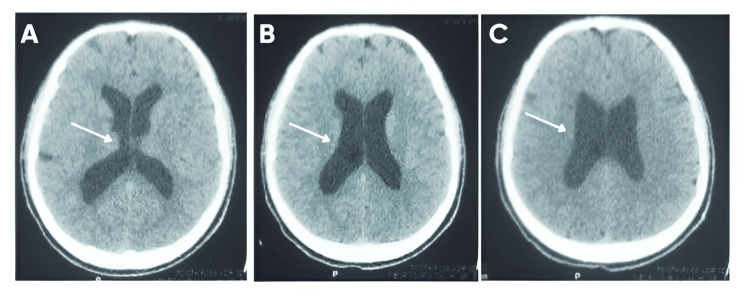
Axial non-contrast CT brain scans demonstrating tri-ventricular hydrocephalus. A: Enlarged frontal horns of the lateral ventricles (white arrows), indicating raised intracranial pressure and narrowing of adjacent sulci. B: Continued dilation of the lateral ventricles (white arrows) and distorted outline of the third ventricle. C: Ballooning of the lateral ventricles (white arrows) with effacement of periventricular structures, consistent with progressive ventricular enlargement.

A previously healthy 17-year-old male with unremarkable past medical history presented with a history of gradually worsening, bilateral blurring of vision during the last three months. The patient was not having any headache, nausea, vomiting, seizure, change in consciousness, or focal neurological deficits. The developmental history was normal, as was the family history for hydrocephalus, intracranial hypertension, or any neurological conditions. On neurological examination, there were no focal motor or sensory deficits. However, ophthalmologic evaluation revealed bilateral papilledema, indicating raised intracranial pressure (ICP). Visual acuity testing showed mild impairment, but visual field examination was normal. A computed tomography (CT) scan of the brain demonstrated tri-ventricular hydrocephalus, with dilation of the lateral and third ventricles while the fourth ventricle remained normal in size (Figure 1).

**Figure 2:**
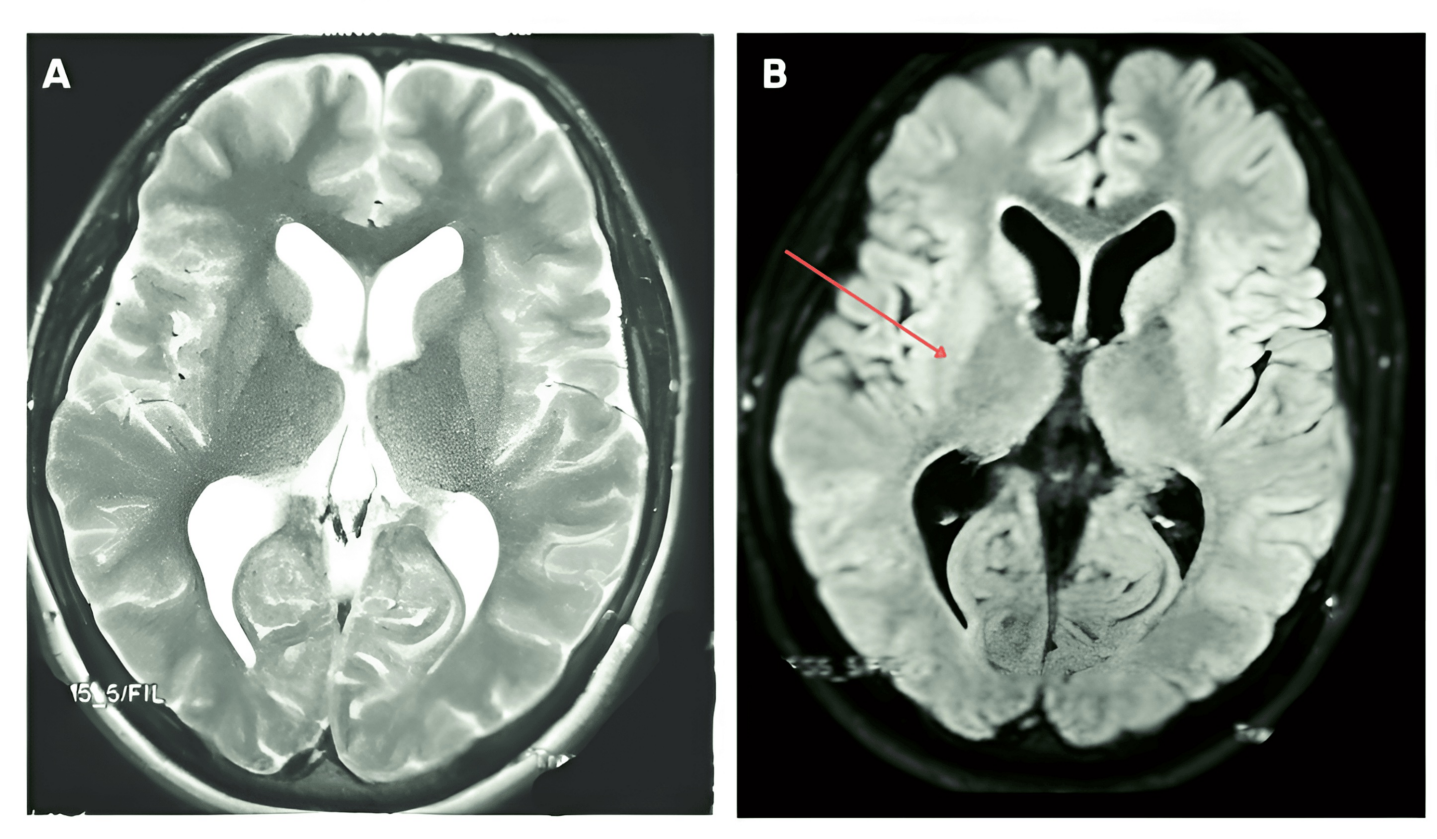
Axial MRI brain sequences demonstrating ventricular enlargement and CSF flow abnormalities. A: Axial T2-weighted MRI shows marked dilatation of the lateral and third ventricles with distortion of the third ventricular floor, consistent with obstructive hydrocephalus. B: Axial fluid-attenuated inversion recovery (FLAIR) MRI reveals persistent ventricular enlargement and periventricular hyperintensity, suggesting CSF absorption dysfunction and evolving intracranial hypertension (red arrow).

No obstructive mass lesions or hemorrhage were noted. Subsequent magnetic resonance imaging (MRI) of the brain, including CSF flow studies, confirmed aqueductal stenosis with absence of CSF flow across the cerebral aqueduct of Sylvius, leading to non-communicating hydrocephalus (Figure 2). After a multidisciplinary discussion, the final diagnosis was established as obstructive hydrocephalus due to congenital aqueductal stenosis. Given the absence of a tumor, cyst, or secondary pathology, the decision was made to proceed with a CSF diversion procedure to restore normal ICP.

The patient underwent an ETV under general anesthesia. A rigid neuroendoscope was introduced via a right frontal burr hole, and the third ventricular floor was identified and perforated using a Fogarty catheter. Adequate CSF egress into the basal cisterns was confirmed intraoperatively. Postoperatively, the patient showed significant improvement in visual symptoms. Follow-up ophthalmologic examination demonstrated gradual resolution of papilledema over the next six weeks.

Two years later, in 2025, at the age of 19, the patient returned with a four-week history of progressively worsening, persistent headaches of moderate intensity. The headache was continuous, worse in the morning, and associated with mild visual blurring. There were no associated symptoms of nausea, vomiting, or focal neurological deficits. A detailed reevaluation was conducted, including a repeat MRI of the brain and magnetic resonance venography (MRV) to assess venous sinus patency. Imaging revealed no evidence of ETV failure, ventricular enlargement, or venous outflow obstruction. However, on ophthalmologic assessment, bilateral grade II papilledema was present.

Given the absence of hydrocephalus and mass lesions, a lumbar puncture with CSF manometry was performed, revealing an elevated opening pressure of 400 mmH₂O, significantly above normal limits. CSF analysis showed normal protein, glucose, and white cell count, ruling out infection, inflammation, or malignancy. Based on these findings, the patient was diagnosed with secondary intracranial hypertension, potentially due to CSF absorption dysfunction. An initial trial of conservative management was attempted using acetazolamide (1 g/day) and serial lumbar punctures to lower CSF pressure. However, the patient continued to experience persistent headaches and papilledema, prompting reconsideration of surgical intervention. Thus, a lumboperitoneal (LP) shunt was indicated for long-term CSF diversion. Under general anesthesia, a lumbar catheter was inserted at the L3-L4 interspace using fluoroscopic guidance. The catheter was attached to a low-pressure valve system and tunneled subcutaneously into the peritoneal space, permitting continuous drainage of CSF. The postoperative course was uneventful, and by 24 hours, the patient was reporting marked symptomatic improvement. Throughout the succeeding weeks, fundoscopy revealed resolution of the papilledema, signifying a successful decrease in CSF pressure. The patient was discharged on postoperative day four with instructions for ongoing neurosurgical and ophthalmologic follow-up.

At his six-month follow-up, the patient remained asymptomatic, with no recurrence of headaches or visual disturbances. Repeat fundoscopy showed persistent resolution of papilledema, and the LP shunt remained functional, with no evidence of over-drainage, infection, or mechanical failure.

## Discussion

This presentation emphasizes the dynamic nature of CSF physiology and the changing pathophysiologic nature of intracranial hypertension disorders in individuals who undergo ETV treatment for obstructive hydrocephalus. Altered vision, specifically papilledema and diplopia, is a key but underestimated presentation of secondary intracranial hypertension caused by aqueductal stenosis. Nakata et al. (2024) [8] described how bilateral papilledema and abducens nerve palsies can also occur in the absence of headache, probably as a consequence of mechanical stretching of the optic and abducens nerves by intermittent intracranial pressure. This corroborates our patient's worsening of vision, resulting in additional diversion of the CSF, even if initially ETV was satisfactory. Also, congenital aqueductal stenosis has a wide range of long-term neuro-ophthalmic complications, usually related to early treatment, as well as the mechanism of secondary hydrocephalus. Smith et al. (2024) [9] highlighted that patients with complex congenital aqueductal stenosis had a significantly higher risk of neurodevelopmental deficits, further emphasizing the need for proactive monitoring of CSF dynamics.

The lack of ventricular dilation on follow-up scans excluded ETV obstruction, prompting a search for secondary causes of increased ICP. MRV also excluded venous outflow obstruction, but CSF manometry documented persistently elevated opening pressures, consistent with a diagnosis of impaired CSF absorption. The phenomenon of intracranial hypertension without ventricular dilation, as seen in this case, aligns with findings by Hansen et al. (1987) [10], who described patients with elevated ICP and impaired CSF absorption but without hydrocephalus. Their study suggested that this paradoxical presentation might be related to altered CSF outflow dynamics and resistance to absorption at the arachnoid granulations despite normal ventricular morphology. Akins and Guppy (2021) [11] also placed emphasis on the impaired glymphatic drainage in disorders of CSF circulation, but they suggested that compromised perivascular clearing functions could be responsible for chronic intracranial hypertension. These findings further underscore the intricacies of CSF homeostasis and indicate the necessity for personalized management in those who are symptomatic while undergoing patent diversion surgery.

For our case, the rationale for undertaking ETV rested on phase-contrast MRI evidence of absent CSF flow through the aqueduct, in line with obstructive hydrocephalus. Clinical improvement in the patient validated the diagnosis and justified ETV as the correct first-line treatment. Even with patent ETV and stable ventricles by imaging, the patient developed recurrent symptomatology as well as papilledema, indicating resolution of the initial obstruction but development of secondary CSF absorption dysfunction.

Our hypothesis is that dysfunctional CSF absorption due to dysfunctional arachnoid granulations or impaired glymphatic clearance resulted in secondary intracranial hypertension in the setting of patent ETV. Our experience is supported by current knowledge about CSF dynamics, which recognizes that one cannot simply say that intracranial pressure is a sole function of ventricular obstruction, but is equally determined by resistance to CSF reabsorption. Anatomical restoration of aqueductal flow by the ETV did not preclude subsequent intracranial hypertension due to increased resistance to CSF outflow, as demonstrated by Olakorede et al. (2025) [12]. Trevisi et al. (2014) [13] further showed there were ETV failures to normalize ICP with patent flow, implicating altered pathways of absorption, potentially through impaired arachnoid granulations or venous sinus function.

The necessity of two separate procedures of CSF diversion in one patient demonstrates the complex nature of hydrocephalus treatment. Park (2022) [14] identifies the fact that, as the gold standard, ventriculoperitoneal (VP) shunting is compromised by complications in terms of infection, dysfunction, and dependency, notably in children. Attempts at decreasing dependency on the shunt include growing use of ETV as a second line of treatment, but this depends on factors unique to the patient, such as age, as well as etiology. Enslin et al. (2021) [15] highlight the global disparities in hydrocephalus care, where access to neuroimaging, follow-up care, and trained neurosurgeons remains limited in low-resource settings, further complicating long-term disease management.

This case highlights that persistent symptoms after a patent ETV may indicate underlying absorptive dysfunction, warranting further CSF pressure studies to guide timely secondary intervention.

## Conclusions

This case highlights the dynamic and evolving nature of CSF disorders. While ETV is an effective first-line intervention for aqueductal stenosis, the subsequent development of intracranial hypertension despite a patent stoma suggests a distinct pathology of CSF absorption dysfunction rather than mechanical obstruction. The need for LP shunting in a previously ETV-treated patient reinforces the necessity for a personalized and adaptive approach to hydrocephalus management. Neurosurgeons must remain vigilant for subtle yet progressive symptoms of elevated ICP, ensuring early intervention to prevent irreversible visual and neurological sequelae. Advances in continuous ICP monitoring, phase-contrast MRI, and individualized CSF diversion strategies will be critical in refining the long-term management of complex CSF circulation disorders.
